# The NLRP3 inflammasome in gynecological cancers: a double-edged sword shaping the immune microenvironment and immunotherapy response

**DOI:** 10.3389/fimmu.2026.1883737

**Published:** 2026-06-24

**Authors:** Yibo He, Shiyue Wu, Zian Shou, Qian Guo, Yaonan Hong, Lianfang Zhao, Jie Wang

**Affiliations:** 1 The First Affiliated Hospital of Zhejiang Chinese Medical University (Zhejiang Provincial Hospital of Chinese Medicine), Hangzhou, Zhejiang, China; 2 School of Pharmaceutical Sciences, Zhejiang Chinese Medical University, Hangzhou, Zhejiang, China; 3 Department of Rhinology, The First Affiliated Hospital of Zhengzhou University, Zhengzhou, China; 4 Department of Medical Genetics, Suining Central Hospital, Suining, Sichuan, China

**Keywords:** gynecological cancer, immune checkpoint, immunotherapy, macrophage polarization, NLRP3 inflammasome, PD-L1, pyroptosis, tumor microenvironment

## Abstract

The NLRP3 inflammasome is a multi-protein innate immune complex that functions as a critical sensor of cellular danger signals, yet its role in gynecological malignancies remains incompletely understood. This mini-review systematically evaluates the paradoxical functions of NLRP3 across the three major gynecological cancers—ovarian, endometrial, and cervical—with emphasis on its dual impact on the tumor immune microenvironment and immunotherapy response. In ovarian cancer, NLRP3 drives immune suppression through PD-L1 upregulation and M2 macrophage polarization via the USP19-STAT6 axis, while simultaneously enhancing cisplatin sensitivity through FTO-mediated pyroptotic signaling. In endometrial cancer, the ERRα-NLRP3-GSDMD pathway regulates pyroptosis in a molecular subtype-dependent manner, with pro-immune effects in MSI-H tumors but potentially pro-tumorigenic consequences in microsatellite-stable subtypes. In cervical cancer, HPV oncoproteins employ multiple mechanisms—including Foxm1-mediated transcriptional suppression, KIF23-dependent GSDMD blockade, and non-coding RNA regulation—to silence NLRP3 and evade immune surveillance. Beyond tumor type-specific mechanisms, the tumor-intrinsic PD-L1/NLRP3 axis has been identified as a key driver of resistance to anti-PD-1 immunotherapy across cancers, whereas NLRP3-activating nanovaccines and small-molecule agonists offer strategies to convert immunologically cold tumors into immunoresponsive ones. We further discuss combination approaches integrating NLRP3 modulators with immune checkpoint inhibitors and PARP inhibitors via the cGAS-STING-NLRP3 axis. Understanding the context-dependent functions of NLRP3 is essential for developing precision immunotherapeutic strategies tailored to tumor type, molecular subtype, and immune context in gynecological malignancies.

## 1 Introduction

Gynecological cancers, including ovarian cancer (OC), endometrial cancer (EC), and cervical cancer (CC), collectively represent a significant global health burden, with approximately 1.3 million new cases and over 600,000 deaths annually (1), and metabolic risk factors including visceral adiposity are increasingly recognized as contributors to cancer-related morbidity (2). Ovarian cancer remains the most lethal gynecological malignancy, with a five-year survival rate below 50% for advanced-stage disease, largely due to late diagnosis and high rates of chemoresistance. Endometrial cancer, while generally associated with favorable outcomes in early-stage disease, exhibits aggressive behavior in advanced or recurrent settings, particularly in the p53-mutant copy number-high molecular subtype. Cervical cancer, driven primarily by persistent high-risk human papillomavirus (HPV) infection, continues to be a leading cause of cancer-related mortality in low- and middle-income countries despite the availability of prophylactic vaccines (3). It is worth distinguishing prophylactic HPV vaccines (which prevent initial infection) from therapeutic HPV-targeted vaccines (which aim to clear established infections and pre-cancerous lesions); the latter conceptually intersect with the NLRP3-activating nanovaccine strategies discussed later in this review.

Despite advances in surgery, chemotherapy, and targeted therapy, the prognosis for patients with advanced or recurrent disease remains poor. Immune checkpoint inhibitors (ICIs) targeting the PD-1/PD-L1 axis have revolutionized cancer treatment across multiple tumor types; however, their efficacy in gynecological malignancies remains limited. Pembrolizumab has received approval for microsatellite instability-high (MSI-H) or mismatch repair-deficient (dMMR) endometrial cancer, yet the overall response rates in ovarian and cervical cancers remain below 15–20% in unselected populations (4, 5). The RUBY and DUO-E trials have demonstrated improved progression-free survival with the addition of dostarlimab or durvalumab to chemotherapy in advanced endometrial cancer, but similar successes have not yet been replicated in ovarian cancer, where the immunosuppressive tumor microenvironment (TME) remains a formidable barrier. This clinical reality underscores the urgent need to better understand the immune heterogeneity within the gynecological TME and to identify novel immunomodulatory targets that can broaden the reach of immunotherapy.

The NLRP3 (NOD-like receptor protein 3) inflammasome is a multi-protein complex that serves as a critical innate immune sensor (6). Classical NLRP3 activation requires a two-signal model: signal 1 (priming) through pattern recognition receptors or cytokine receptors, which upregulates NLRP3 and pro-IL-1β transcription via NF-κB signaling; and signal 2 (activation) triggered by diverse danger signals including ATP, crystalline substances, and mitochondrial reactive oxygen species (ROS) (7). Upon activation, NLRP3 recruits the adaptor protein ASC and pro-caspase-1, leading to caspase-1 auto-activation. Active caspase-1 subsequently processes pro-IL-1β and pro-IL-18 into their mature forms and cleaves gasdermin D (GSDMD), whose N-terminal domain forms membrane pores that trigger pyroptosis (8). Beyond the classical pathway, non-canonical NLRP3 activation via caspase-4/5/11 has been increasingly recognized in cancer biology (9).

**Figure 1:**
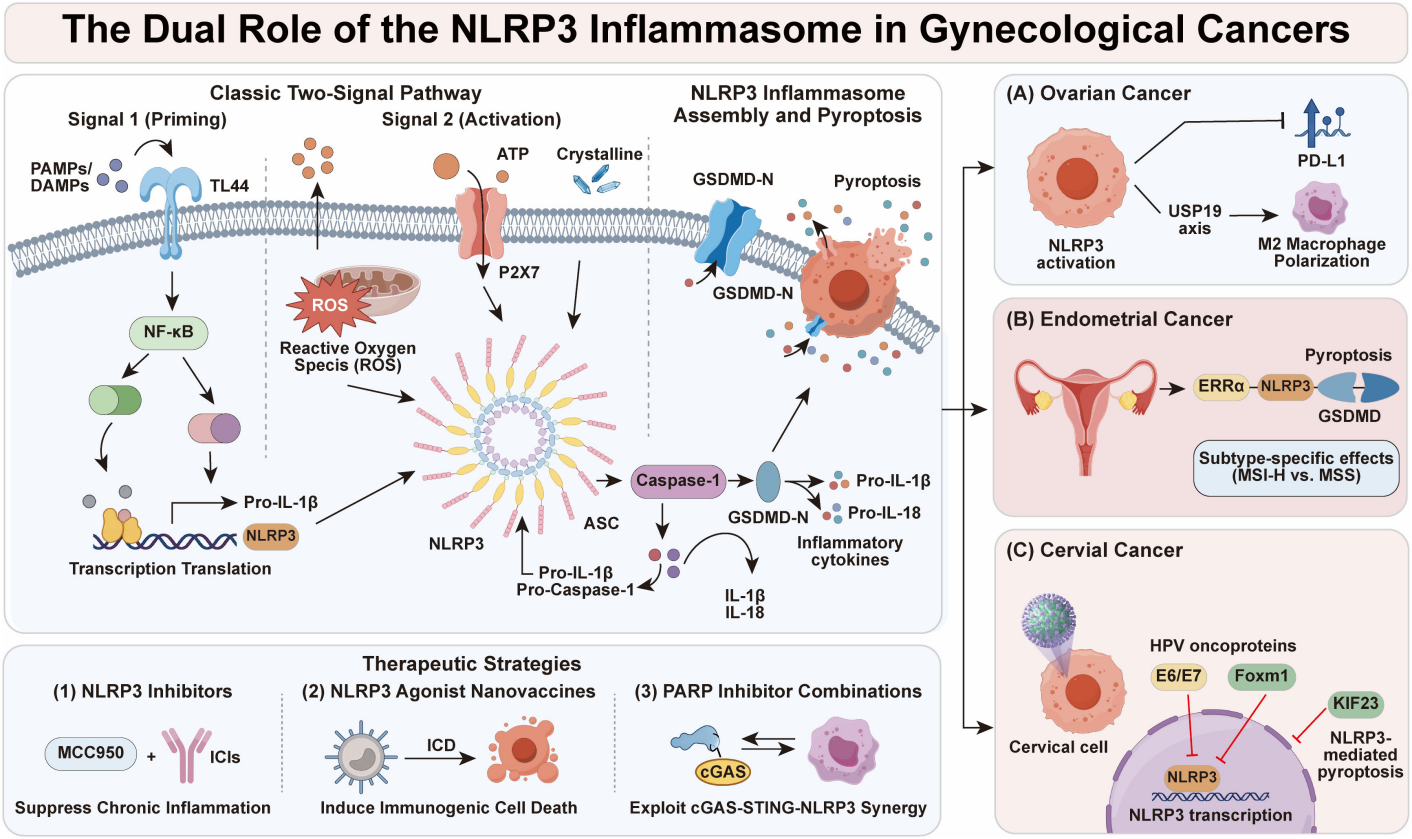
The dual role of NLRP3 inflammasome in gynecological cancers. This figure illustrates the dual role of the NLRP3 inflammasome in gynecological cancers, which is activated by NF-κB priming (Signal 1) and danger signals like ATP and ROS (Signal 2), leading to GSDMD-mediated pyroptosis and cytokine release. The mechanisms vary across specific cancers: **(A)** in ovarian cancer, NLRP3 promotes immune evasion via PD-L1 and M2 macrophage polarization while enhancing cisplatin sensitivity through pyroptosis; **(B)** in endometrial cancer, the ERRα-NLRP3-GSDMD axis regulates subtype-specific pyroptosis in MSI-H versus MSS tumors; and **(C)** in cervical cancer, HPV oncoproteins and factors like Foxm1 and KIF23 suppress NLRP3 to facilitate tumor progression. Pharmacological nodes are annotated directly on the pathway: NLRP3 inhibitors (MCC950, OLT1177) block inflammasome assembly; NLRP3 agonists (nigericin, ATP, nanovaccine-encapsulated activators) promote pyroptosis and ICD; STING agonists (ADU-S100, MK-1454) engage the cGAS-STING-NLRP3 crosstalk; and PARP inhibitors (olaparib, niraparib) activate cGAS-STING via micronuclei formation. Clinical strategies include using NLRP3 inhibitors to overcome ICI resistance, nanovaccines for tumor-localized ICD induction, and rational combination approaches guided by tumor NLRP3 activation status and molecular subtype.

The role of NLRP3 inflammasome in cancer is highly context-dependent and often paradoxical (10). On one hand, NLRP3-mediated IL-1β and IL-18 secretion can promote chronic inflammation, angiogenesis, and immune suppression, thereby facilitating tumor progression (11). On the other hand, NLRP3-driven pyroptosis represents a form of immunogenic cell death (ICD) that can release damage-associated molecular patterns (DAMPs) and activate anti-tumor immunity (12). It is important to note that the immunogenicity of pyroptosis is not solely attributable to the cell death modality itself, but critically depends on the concomitant release of IL-1β and IL-18, which serve as maturation signals for dendritic cells and promote CD8+ T cell priming. Distinguishing NLRP3-dependent cytokine release from the lytic consequences of GSDMD pore formation is methodologically challenging but conceptually important for rational drug design. This dual nature is particularly relevant in gynecological cancers, where the TME is characterized by profound immune heterogeneity. This mini-review aims to systematically evaluate the role of NLRP3 in OC, EC, and CC, with emphasis on its impact on the immune landscape and implications for immunotherapy (**Figure 1**, **Table 1**).

**Table 1:** Key studies on NLRP3 inflammasome in gynecological cancers.

Cancer type	Key finding	Mechanism	Reference
Ovarian	Single-cell decoding of inflammatory regulation	scRNA-seq reveals cell type-specific inflammatory signatures	(14)
Ovarian	NLRP3 upregulates PD-L1 expression	NF-κB/MAPK-mediated PD-L1 transcription; immune suppression	(15)
Ovarian	USP19 suppresses NLRP3 to promote M2 polarization	STAT6 stabilization; M2 macrophage reprogramming	(18)
Ovarian	FTO triggers NLRP3/GSDMD pyroptosis	m6A demethylation of NLRP3 mRNA; enhanced cisplatin sensitivity	(19)
Ovarian	Necroptosis-associated lncRNAs predict cold/hot tumors	Programmed cell death network shapes immune landscape	(21)
Ovarian	Ferroptosis causally linked to ovarian dysfunction	MR integrative multi-omics (DNA methylation, proteome)	(22)
Ovarian	Inflammasome activation in cisplatin resistance	Chronic IL-1β/NF-κB pro-survival signaling	(23)
Endometrial	ERRα targets NLRP3/caspase-1/GSDMD pathway	HIF-1α-dependent glycolysis suppresses NLRP3	(25)
Endometrial	H_2_ inhibits cancer growth via NLRP3 pyroptosis	ROS-mediated NLRP3/caspase-1/GSDMD activation	(26)
Cervical	Foxm1 inactivates NLRP3 transcription	Transcriptional suppression; immune evasion	(31)
Cervical	KIF23 inhibits NLRP3-mediated pyroptosis	Suppression of GSDMD cleavage; tumor progression	(32)
Cervical	NLRP3 activation in peripheral monocytes	IL-1β release; correlation with survival	(33)
Cervical	NLRP3 gene polymorphisms (rs10754558)	Genetic susceptibility to HPV and cervical cancer	(34)
Cervical	LRRC75A-AS1 inhibits NLRP3 ubiquitination	IGF2BP1 binding; EMT promotion	(37)
Pan-cancer	NLRP3 correlates with immune checkpoints	PD-L1, CTLA-4, LAG-3 co-expression	(16)
Pan-cancer	Tumor-intrinsic PD-L1/NLRP3 drives ICI resistance	Autocrine NLRP3 signaling; anti-PD-1 resistance	(38)
Pan-cancer	Small molecule targets NLRP3 for antitumor immunity	NLRP3 agonism promotes ICD	(42)

## 2 NLRP3 inflammasome in ovarian cancer

Ovarian cancer is characterized by a highly immunosuppressive TME dominated by tumor-associated macrophages (TAMs), regulatory T cells (Tregs), and myeloid-derived suppressor cells (MDSCs) (13). The peritoneal cavity, which is the primary site of metastatic spread in high-grade serous ovarian cancer (HGSOC), creates a unique inflammatory milieu enriched with cytokines, chemokines, and ascitic fluid that collectively promote immune evasion. Recent single-cell transcriptomic analyses have decoded the inflammatory regulatory networks in ovarian cancer at single-cell resolution, identifying cell type-specific inflammatory signatures that correlate with clinical outcomes and revealing extensive heterogeneity in immune cell states across patients (14). These studies have revealed a critical link between NLRP3 inflammasome activation and the establishment of this immunosuppressive milieu, positioning NLRP3 as a key node connecting chronic inflammation with immune escape in ovarian cancer.

Framework for this section: We address NLRP3 in ovarian cancer through the following lenses: (a) NLRP3 expression pattern and correlation with clinical features; (b) functional evidence for pro-tumor vs. anti-tumor roles and key signaling intermediates; (c) relevance to standard-of-care platinum-based chemotherapy; (d) implications for ICI response; and (e) outstanding questions. This structure is paralleled in Sections 3 and 4 to facilitate cross-tumor comparison.

### 2.1 NLRP3-PD-L1 axis and macrophage polarization

A landmark study by Pan et al. demonstrated that NLRP3 inflammasome activation directly upregulates PD-L1 expression in ovarian cancer cells (15). Mechanistically, NLRP3-dependent IL-1β secretion activates NF-κB and MAPK signaling in an autocrine and paracrine manner, leading to transcriptional upregulation of PD-L1. This creates a positive feedback loop: NLRP3 activation drives inflammation while simultaneously suppressing CD8+ T cell effector function through PD-1/PD-L1 engagement. Importantly, this axis operates independently of interferon-γ signaling, which has traditionally been considered the primary driver of PD-L1 expression in the TME. The finding is particularly relevant in ovarian cancer, where PD-L1 expression is heterogeneous and poorly predicts response to anti-PD-1 monotherapy. Ding et al. extended these observations through a pan-cancer analysis revealing that NLRP3 expression positively correlates with PD-L1, CTLA-4, and LAG-3 across multiple cancer types, and that high NLRP3 is associated with elevated Treg infiltration and reduced CD8^+^ T cell cytotoxicity (16). These results position NLRP3 as an upstream regulator of immune checkpoint expression.

Beyond tumor cell-intrinsic effects, NLRP3 also shapes the immune landscape through macrophage polarization. TAMs are predominantly skewed toward the immunosuppressive M2 phenotype in ovarian cancer, promoting tumor growth through IL-10, TGF-β, and VEGF secretion while suppressing CD8^+^ T cell activity via arginase-1 and IDO expression (17). Zhang et al. identified ubiquitin-specific protease 19 (USP19) as a key negative regulator of NLRP3 that promotes M2 polarization via deubiquitination and stabilization of STAT6, which suppresses NLRP3 expression (18). Conversely, NLRP3 activation in macrophages favors M1 polarization and production of TNF-α, IL-6, and IL-12, enhancing antigen presentation and CD8+ T cell priming. This USP19-STAT6-NLRP3 axis suggests that pharmacological USP19 inhibition could reprogram TAMs and restore anti-tumor immunity.

### 2.2 NLRP3-mediated pyroptosis and chemotherapy response

NLRP3-mediated pyroptosis has emerged as a critical determinant of chemotherapy sensitivity. Zhang et al. demonstrated that the RNA demethylase FTO triggers NLRP3/GSDMD-dependent pyroptosis to enhance cisplatin sensitivity through m6A demethylation and stabilization of NLRP3 mRNA (19). Pyroptotic cell death releases DAMPs including HMGB1, ATP, and calreticulin, which activate dendritic cells and promote CD8^+^ T cell infiltration (20). Beyond pyroptosis, other forms of regulated cell death contribute to the ovarian cancer immune landscape: necroptosis-associated lncRNAs predict prognosis and differentiate between immunologically cold and hot tumors (21), and ferroptosis—mechanistically linked to NLRP3 through iron metabolism and ROS generation—has been causally associated with ovarian dysfunction (22). These findings suggest that combining cisplatin with NLRP3-activating agents could enhance immunogenic chemotherapy responses.

The relationship is not straightforward, however. De Souza et al. showed that chronic inflammasome activation can also contribute to cisplatin resistance through sustained IL-1β signaling and NF-κB-mediated pro-survival gene expression (23). In their model, prolonged NLRP3 activation promoted an anti-apoptotic phenotype in ovarian cancer cells, counteracting the pro-death effects of acute inflammasome engagement. These contrasting effects underscore the importance of temporal and spatial regulation of NLRP3—acute activation may sensitize tumors to chemotherapy, whereas chronic activation may fuel resistance.

Outstanding questions for ovarian cancer: (1) What threshold of NLRP3 activation distinguishes cisplatin sensitization from cisplatin resistance? (2) Can the FTO-NLRP3-GSDMD axis be pharmacologically engaged in tumors with low basal NLRP3 expression? (3) Do platinum-resistant tumors exhibit fundamentally different NLRP3 pathway configurations compared to platinum-sensitive tumors, and can these be therapeutically reversed?

## 3 NLRP3 inflammasome in endometrial cancer

The molecular classification of endometrial cancer into POLE ultramutated, MSI-H, copy number-low (CN-low), and copy number-high (CN-high) subtypes carries important immunological implications (24). TCGA analysis revealed that immune contexture varies dramatically across these subtypes: POLE tumors harbor the highest tumor mutational burden (TMB) and neoantigen load, MSI-H tumors display prominent lymphocytic infiltration and have shown the greatest sensitivity to ICIs in the RUBY and GY018 trials, while CN-high tumors are largely immunologically “cold.” CN-low tumors occupy an intermediate position, displaying moderate immune infiltration. This molecular heterogeneity creates distinct immune landscapes in which NLRP3 may play divergent roles, a concept that is only beginning to be explored in the literature.

Framework for this section: We address NLRP3 in endometrial cancer through: (a) NLRP3 and downstream effector expression across molecular subtypes; (b) functional evidence for pro-tumor vs. anti-tumor roles; (c) relevance to chemotherapy and hormonal therapy; (d) implications for ICI response by subtype; and (e) outstanding questions.

### 3.1 NLRP3-GSDMD pyroptotic pathway

Su et al. provided the first comprehensive characterization of the NLRP3 pyroptotic pathway in endometrial cancer, demonstrating that the transcription factor ERRα promotes glycolytic metabolism while simultaneously targeting the NLRP3/caspase-1/GSDMD pathway to regulate pyroptosis (25). ERRα activates HIF-1α-dependent glycolysis, which paradoxically suppresses NLRP3 inflammasome assembly through metabolite-mediated inhibition. This metabolic-immune crosstalk represents a novel mechanism by which endometrial cancer cells evade pyroptotic death, linking the Warburg effect to innate immune evasion. The clinical significance of this axis is further underscored by the fact that ERRα and HIF-1α are both commonly upregulated in advanced endometrial cancer, suggesting that metabolic suppression of NLRP3 may be a widespread phenomenon rather than an isolated observation. In a separate study, hydrogen gas was shown to inhibit endometrial cancer growth through a ROS/NLRP3/caspase-1/GSDMD-mediated pyroptotic pathway, confirming the tumor-suppressive potential of NLRP3 activation in this context (26). These findings highlight that metabolic reprogramming in endometrial cancer directly intersects with NLRP3-mediated immune signaling, and suggest that agents capable of disrupting the ERRα-HIF-1α axis could restore NLRP3-dependent tumor suppression.

### 3.2 Subtype-dependent roles of NLRP3

The functional consequences of NLRP3 activation differ markedly across molecular subtypes, a consideration that has important implications for immunotherapy trial design. In MSI-H endometrial cancers, the elevated TMB generates abundant neoantigens that, when combined with NLRP3-driven immunogenic cell death, may potentiate anti-tumor immune responses (27). This synergy may partly explain the superior ICI responses observed in MSI-H patients. In contrast, microsatellite-stable (MSS) tumors lack sufficient neoantigen presentation to capitalize on pyroptosis-induced immunity, and the released IL-1β and IL-18 may instead promote tumor progression through STAT3 activation and angiogenesis (28). Consistent with this model, emerging evidence indicates that high NLRP3 expression correlates with increased CD8+ T cell and NK cell infiltration in POLE and MSI-H tumors, whereas in CN-high tumors, NLRP3 activation may favor immunosuppressive myeloid recruitment and Treg expansion (16). These observations underscore that NLRP3-directed strategies should be tailored to molecular subtype rather than applied uniformly—a principle that aligns with the broader movement toward precision oncology in gynecological cancers.

Outstanding questions for endometrial cancer: (1) Does NLRP3 activation status correlate with response to hormonal therapy (e.g., progestins, aromatase inhibitors) in MSS tumors? (2) Can pharmacological NLRP3 activation synergize with ICI therapy specifically in the MSS/CN-low subgroup to convert these tumors from immunologically intermediate to immunologically hot? (3) What is the relationship between NLRP3 pathway activity and the efficacy of PARP inhibitors in the ~5–10% of endometrial cancers harboring BRCA1/2 or HRD mutations?

## 4 NLRP3 inflammasome in cervical cancer

Cervical cancer is primarily driven by persistent high-risk HPV infection, particularly HPV16 and HPV18, which together account for approximately 70% of all cases (29). Accurate nodal staging remains critical for prognosis and treatment planning in cervical cancer (30). The HPV oncoproteins E6 and E7 drive carcinogenesis through degradation of p53 and inactivation of Rb, respectively. Beyond these canonical functions, viral proteins actively subvert innate immune signaling to establish persistent infection. The interplay between HPV oncoproteins and NLRP3 has emerged as a critical area of investigation, as understanding this crosstalk may reveal strategies to overcome immune resistance.

Framework for this section: We address NLRP3 in cervical cancer through: (a) NLRP3 expression patterns in HPV-positive vs. HPV-negative disease; (b) mechanisms of HPV-mediated NLRP3 suppression; (c) relevance to cisplatin-based chemoradiation (the standard of care for locally advanced disease); (d) implications for ICI response (pembrolizumab is approved for PD-L1-positive recurrent/metastatic CC); and (e) outstanding questions.

### 4.1 HPV-driven NLRP3 suppression and regulatory networks

HPV oncoproteins modulate NLRP3 activity through multiple, partly redundant mechanisms. Wang et al. reported that Foxm1, a forkhead box transcription factor frequently overexpressed in HPV-positive cervical cancers due to E7-mediated Rb inactivation, directly binds to the NLRP3 promoter and suppresses its transcription, thereby reducing IL-1β and IL-18 secretion and impairing anti-tumor immunity (31). This transcriptional silencing represents a fundamentally different strategy from post-translational NLRP3 regulation observed in other cancer types. Independently, KIF23—a kinesin family member upregulated in cervical cancer—inhibits NLRP3-mediated pyroptosis by blocking GSDMD cleavage at a post-activation step, promoting tumor cell survival and immune evasion (32). Together, these mechanisms reveal that cervical cancer cells employ multiple layers of NLRP3 suppression—both transcriptional and post-translational—to maintain an immunologically silent state.

Non-coding RNAs add further complexity to this regulatory landscape and may represent therapeutic targets in their own right. MiR-214 promotes pyroptosis and inhibits cervical cancer cell proliferation through NLRP3 upregulation; overexpression of miR-214 enhanced caspase-1 activation and GSDMD cleavage, leading to increased pyroptotic cell death and reduced colony formation (33). Conversely, the lncRNA LRRC75A-AS1 drives EMT by binding IGF2BP1 and inhibiting SYVN1-mediated NLRP3 ubiquitination, paradoxically stabilizing NLRP3 protein while promoting tumor invasion rather than immune activation (34). This finding is notable because it demonstrates that NLRP3 stabilization does not always translate to anti-tumor immunity—the downstream signaling context determines whether activation leads to immunogenic pyroptosis or pro-metastatic signaling, a principle also observed in pan-cancer analyses of EMT regulators in the TME (35).

### 4.2 NLRP3 activation and immune biomarkers

Despite the suppressive mechanisms employed by tumor cells, NLRP3 can also be activated in the cervical cancer microenvironment, with important prognostic implications. Mendonça et al. demonstrated that cervical carcinoma induces NLRP3 inflammasome activation and IL-1β release in peripheral blood monocytes, which positively correlates with patient overall survival (36). This paradoxical finding suggests that systemic NLRP3 activation in immune cells may reflect an intact anti-tumor immune response and could serve as a prognostic biomarker. Patients with higher monocyte-derived IL-1β production exhibited superior survival outcomes, indicating that NLRP3 activation in immune cells—rather than tumor cells—may be associated with favorable prognosis. Furthermore, NLRP3 gene polymorphisms (rs10754558 and rs10733113) have been associated with susceptibility to HPV infection and cervical cancer development in certain populations (37). The rs10754558 polymorphism, located in the 3’-UTR of NLRP3, affects mRNA stability and has been linked to altered NLRP3 expression levels, indicating a genetic predisposition component that may influence individual immune responses to HPV infection and cervical carcinogenesis.

Outstanding questions for cervical cancer: (1) Can pharmacological reversal of Foxm1-mediated or KIF23-mediated NLRP3 suppression restore cisplatin sensitivity in chemoradiation-resistant disease? (2) Does NLRP3 activation status in pre-treatment biopsies predict response to pembrolizumab in the recurrent/metastatic setting? (3) Given that NLRP3 is silenced by multiple redundant HPV-driven mechanisms, is single-agent NLRP3 de-repression sufficient, or will combination strategies targeting multiple nodes be required?

## 5 Therapeutic implications: targeting NLRP3 in gynecological cancers

The dual role of NLRP3 in gynecological cancers—simultaneously driving immune suppression and immunogenic cell death—necessitates carefully designed therapeutic strategies that account for tumor type, molecular subtype, and the dominant immune context within each tumor.

### 5.1 NLRP3 as a driver of immunotherapy resistance

The tumor-intrinsic PD-L1/NLRP3 axis has been identified as a key driver of anti-PD-1 resistance (38). Theivanthiran et al. demonstrated that tumor cell-intrinsic PD-L1 activates NLRP3 signaling, promoting immunosuppressive factor production in a self-reinforcing manner. PD-L1 engagement with PD-1 on CD8+ T cells triggers intracellular signaling that activates the NLRP3 inflammasome in tumor cells, leading to IL-1β secretion that further dampens anti-tumor immunity. This finding has particular clinical relevance, as it identifies a mechanism of resistance that operates downstream of PD-1/PD-L1 blockade.

Targeting this pathway with NLRP3 inhibitors such as MCC950 has shown promise in restoring ICI sensitivity in preclinical models (39). The NLRP3-HSP70 axis has also been implicated in resistance: HSP70, released during cellular stress and NLRP3 activation, promotes dendritic cell tolerization and Treg expansion, and pharmacological inhibition of this axis overcame anti-PD-1 resistance in preclinical cancer models (40). These dual signaling nodes—the PD-L1/NLRP3 and NLRP3/HSP70 axes—provide complementary therapeutic targets for overcoming immunotherapy resistance.

### 5.2 Harnessing pyroptosis for immunotherapy

Conversely, NLRP3-mediated pyroptosis offers a strategy to convert immunologically “cold” tumors into “hot” ones. Wang et al. provided direct evidence that pyroptotic tumor cells elicit potent anti-tumor immune responses using a bioorthogonal chemistry approach ^9^. Building on this concept, Zhen et al. developed inflammasome-activating nanovaccines that trigger NLRP3-dependent ICD specifically within tumors while sparing normal tissues from excessive inflammation (41), and Hu et al. identified a small molecule that directly targets NLRP3 to promote inflammasome activation and antitumor immunity (42). These approaches are particularly relevant for ovarian and cervical cancers, where the TME is typically immunologically cold and intraperitoneal or intratumoral delivery routes are clinically feasible.

### 5.3 Combination strategies

Several combination approaches are under investigation. MCC950 or next-generation NLRP3 inhibitors combined with anti-PD-1/PD-L1 antibodies aim to suppress chronic inflammation while enhancing T cell function (39). Alternatively, tumor-localized NLRP3 activation via nanovaccine delivery could induce ICD and synergize with checkpoint blockade, particularly in immunologically cold tumors. Notably, PARP inhibitor-induced DNA damage activates the cGAS-STING pathway, which can prime NLRP3 assembly—suggesting synergistic potential in BRCA-mutant ovarian cancers (43). The mechanistic relationship between cGAS-STING and NLRP3 is bidirectional and context-specific: in myeloid cells, STING activation upstream of NLRP3 can promote inflammasome assembly through lysosomal disruption and K^+^ efflux, whereas in tumor cells, NLRP3-derived mitochondrial damage can secondarily engage cGAS-STING via release of mitochondrial DNA into the cytosol. This bidirectional crosstalk has important therapeutic implications: STING agonists (e.g., ADU-S100, MK-1454, and next-generation non-cyclic dinucleotide agonists) may inadvertently exacerbate NLRP3-driven immunosuppression if applied in tumors with pre-existing NLRP3 hyperactivity, while conversely potentiating anti-tumor immunity in NLRP3-low tumors. The clinical development of systemic STING agonists has been hampered by dose-limiting inflammatory toxicities, including fever, cytokine release syndrome, and vascular leakage; tumor-targeted delivery strategies—such as intratumoral injection, liposomal encapsulation, or antibody-drug conjugates—are being explored to mitigate these risks. The cGAS-STING-NLRP3 axis also converges on type I interferon (IFN) responses: cGAS-STING activation induces IFN-β via TBK1-IRF3, while NLRP3 can both potentiate (through IL-1β-mediated amplification) and suppress (through caspase-1-mediated degradation of cGAS) this response. In BRCA-mutant ovarian cancers, PARP inhibitor-induced micronuclei activate cGAS-STING, providing a mechanistic rationale for triple combination strategies involving PARP inhibitors, ICIs, and NLRP3 modulators, with the choice of NLRP3 inhibitor vs. agonist depending on whether the dominant NLRP3 function in that context is immunosuppressive or immunostimulatory.

Recent advances in STING agonist development have further expanded this therapeutic landscape, with next-generation activators showing enhanced tumor selectivity and favorable pharmacokinetic profiles (44). This cGAS-STING-NLRP3 axis may explain the enhanced immunotherapy responses observed in HRD-positive tumors and provides a molecular rationale for triple combination strategies involving PARP inhibitors, ICIs, and NLRP3 modulators.

The choice between NLRP3 inhibition and activation ultimately depends on the dominant immune context: inhibition for tumors with chronic NLRP3-driven inflammation and elevated immunosuppressive cytokines, and activation for immunologically cold tumors with sparse immune infiltration. Companion diagnostic approaches that assess NLRP3 pathway activation status, cytokine profiles, and immune cell composition may be essential for optimal patient selection in future clinical trials. In this regard, multiplex immunofluorescence and spatial transcriptomics could provide the spatial resolution needed to determine whether NLRP3 is predominantly active in tumor cells or stromal immune cells within a given tumor.

### 5.4 Pharmacological toolbox: NLRP3 small molecule modulators

The translational promise of NLRP3-targeted therapy in gynecological cancers depends critically on the availability of selective, drug-like small molecules. Below we provide a systematic overview of the key NLRP3 modulators with demonstrated relevance to cancer biology (**Table 2**).

**Table 2:** Key NLRP3 small molecule modulators with relevance to cancer therapy.

Compound	Mechanism	IC_50_/potency	Selectivity	Development stage	Cancer data	Key limitation
MCC950 (CRID3)	NACHT domain binding; blocks ATP hydrolysis	IC_50_ ~7.5 nM (inflammasome)	NLRP3-selective; spares NLRC4, AIM2	Preclinical	Reverses anti-PD-1 resistance	High protein binding; short t_1_/_2_; carbonic anhydrase off-target
CY-09	Walker A motif binding; blocks ATP binding	IC_50_ ~500 nM	NLRP3-selective	Preclinical	No gynecological cancer data	Limited PK characterization *in vivo*
OLT1177 (dapansutrile)	NLRP3 ATPase inhibition	IC_50_ ~100 nM	NLRP3-selective	Phase II (OA, HF)	No oncology data published	Unstudied in tumor models
Tranilast	NACHT domain binding	IC_50_ ~5–10 μM	Multi-target	Clinically approved (allergy)	No cancer-specific studies	Low potency; poor selectivity
BAY 11-7082	NLRP3 ATPase + NF-κB inhibition	IC_50_ ~1–5 μM	Non-selective	Preclinical tool compound	Not suitable for target validation	Dual pharmacology confounds interpretation
Nigericin	K^+^ ionophore; NLRP3 activator	EC_50_ ~1–10 μM	Broad inflammasome activator	Research tool only	Preclinical ICD induction	Systemic toxicity; not drug-like
Novel NLRP3 agonist	Direct NLRP3 engagement (mechanism TBD)	Not fully characterized	Reportedly NLRP3-selective	Early preclinical	Antitumor immunity	Limited selectivity profiling; no PK data

#### 5.4.1 NLRP3 inhibitors

MCC950 (CRID3) is the prototypical NLRP3 inhibitor, acting through direct binding to the NACHT domain and blocking ATP hydrolysis, thereby preventing NLRP3 oligomerization and inflammasome assembly. MCC950 has shown efficacy in reversing anti-PD-1 resistance in preclinical models and in abrogating ICI-related cardiotoxicity (39). However, its clinical translatability is limited by several pharmacokinetic liabilities: high plasma protein binding (>99%), short half-life *in vivo*, and off-target effects on carbonic anhydrase at micromolar concentrations. These limitations have spurred the development of next-generation inhibitors.

CY-09 directly binds to the Walker A motif of NLRP3 and inhibits ATP binding, showing efficacy in metabolic disease models. Its selectivity profile and pharmacokinetic properties in tumor-bearing models remain to be fully characterized.

OLT1177 (dapansutrile) is an orally active β-sulfonyl nitrile compound that inhibits NLRP3 ATPase activity. It has completed Phase II clinical trials for osteoarthritis and heart failure, demonstrating a favorable safety profile. Its evaluation in oncology indications, including potential combination with ICIs, represents an attractive near-term translational opportunity.

Tranilast, an older anti-allergic drug, was repurposed as an NLRP3 inhibitor through direct binding to the NACHT domain. Its established clinical safety record and oral bioavailability make it a candidate for rapid repurposing, though its potency (micromolar range) and lack of target selectivity warrant caution.

BAY 11–7082 inhibits NLRP3 ATPase activity but also targets NF-κB, limiting its utility as a probe for NLRP3-specific pharmacology.

#### 5.4.2 NLRP3 activators

Nigericin, a bacterial ionophore, is the most widely used experimental NLRP3 activator, acting through K^+^ efflux. Its use is confined to preclinical studies due to systemic toxicity.

ATP at millimolar concentrations activates the P2X7 receptor, triggering K^+^ efflux and NLRP3 assembly. Extracellular ATP is abundant in the tumor microenvironment due to cell death and metabolic stress, providing an endogenous NLRP3 activation mechanism that may be therapeutically exploitable.

MSU (monosodium urate) crystals and alum activate NLRP3 through lysosomal damage and are used primarily as adjuvants in vaccine formulations. Their relevance to NLRP3-activating nanovaccine design (41) is discussed in Section 5.2.

Novel small-molecule NLRP3 agonists identified through phenotypic screening represent an emerging class. One such compound was recently shown to directly engage NLRP3 and promote antitumor immunity (42), though target engagement data and selectivity profiling remain limited.

#### 5.4.3 Key considerations for clinical translation

Several pharmacological considerations are particularly relevant to gynecological cancers. First, intraperitoneal delivery of NLRP3 modulators may offer pharmacokinetic advantages in ovarian cancer with peritoneal involvement, achieving high local drug concentrations while limiting systemic exposure. Second, the blood-brain barrier penetration of NLRP3 inhibitors is generally low, which may be advantageous for oncology indications by reducing CNS-mediated toxicity. Third, tumor cell-intrinsic vs. immune cell-intrinsic NLRP3 targeting requires different drug properties: tumor cell targeting benefits from enhanced tissue penetration and intracellular retention, whereas immune cell targeting may be adequately served by compounds with more favorable systemic distribution. Fourth, the temporal dynamics of NLRP3 modulation—pulsatile vs. continuous target engagement—may influence whether the net effect is immunostimulatory or immunosuppressive, a consideration that has received insufficient attention in the preclinical literature.

Several critical knowledge gaps remain that warrant focused investigation. First, single-cell multi-omics approaches are needed to resolve cell type-specific roles of NLRP3 within the heterogeneous TME, distinguishing tumor cell-intrinsic signaling from activation in immune cells. Current bulk transcriptomic analyses cannot adequately capture this functional heterogeneity across different cell populations within the same tumor. Second, the temporal dynamics of NLRP3 activation during disease progression, chemotherapy, and immunotherapy remain poorly understood. Longitudinal profiling of NLRP3 pathway components in matched pre- and post-treatment samples could reveal how NLRP3 function evolves under therapeutic pressure—shifting from tumor-suppressive to tumor-promoting or vice versa as the TME remodels. Third, NLRP3 may serve as a biomarker for immunotherapy response prediction when combined with established markers such as MSI status, TMB, and PD-L1 expression, enabling more precise patient stratification.

Looking ahead, two directions are particularly promising. Targeting ferroptosis, which shares mechanistic crosstalk with NLRP3 through iron metabolism and ROS signaling (45), represents a complementary therapeutic strategy for gynecological disorders and cancers (46, 47). Understanding how ferroptosis and pyroptosis cooperatively shape the immune microenvironment may reveal combination strategies that simultaneously engage multiple immunogenic cell death pathways. Equally important is the development of tumor-targeted NLRP3 modulators—agonists for ICD induction in immunologically cold tumors, or inhibitors for chronic inflammation suppression in hot tumors. Translating these mechanistic insights into precision immunotherapy approaches will be essential for improving outcomes in gynecological malignancies.

## 6 Future perspectives

### 6.1 Cell-type specificity of NLRP3 function in the TME

A critical gap in the current literature is the lack of a systematic understanding of the cell type-specific functions of NLRP3 within the gynecological TME. The therapeutic outcome of NLRP3 modulation is likely to depend on its distinct roles across tumor, immune, and stromal cell populations.

**Figure 2:**
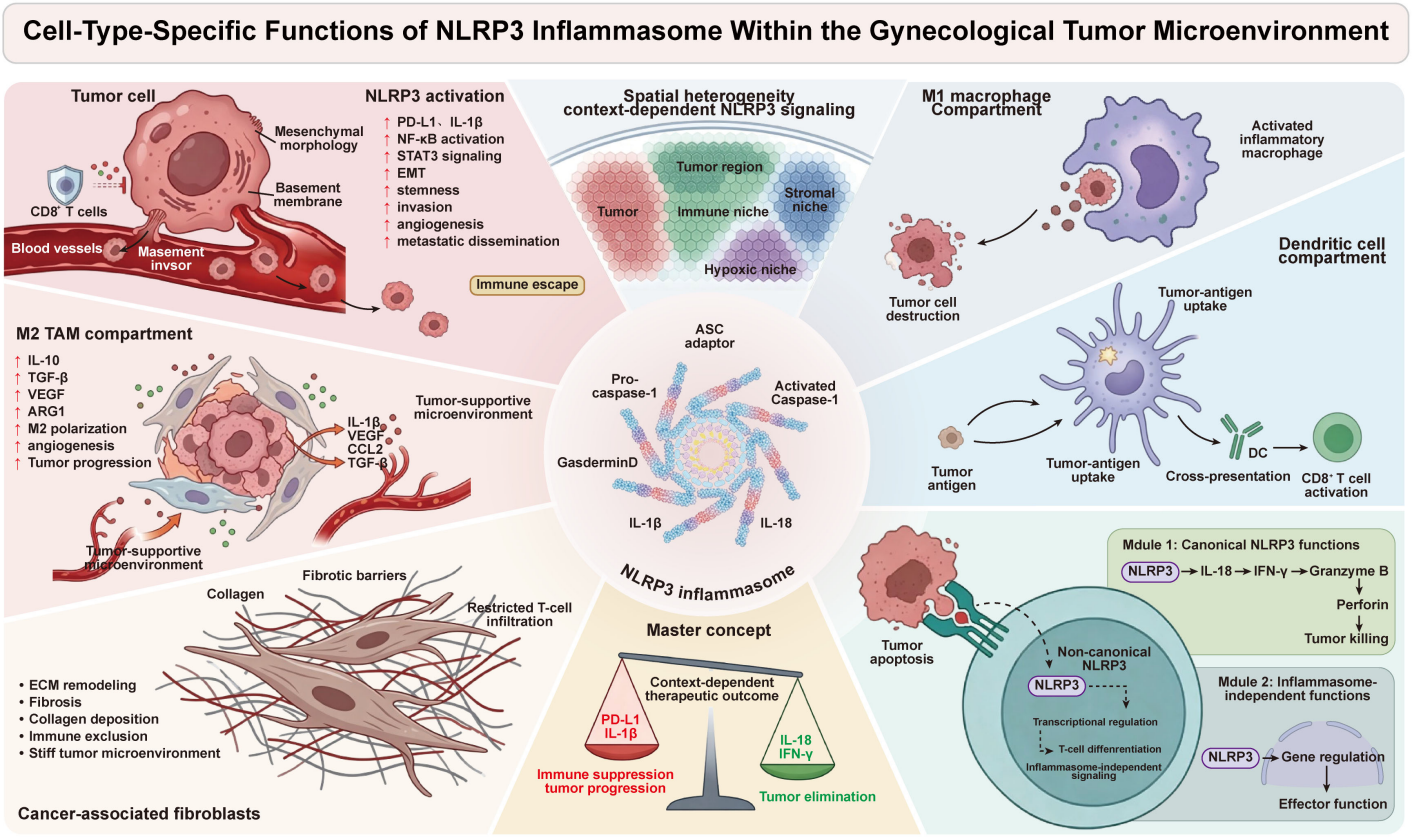
Cell-type-specific functions of the NLRP3 inflammasome in the gynecological tumor microenvironment. This figure illustrates the compartment-specific and often opposing roles of NLRP3 across the major cell populations of the gynecological TME. In tumor cells, NLRP3 drives both PD-L1-mediated immune evasion and GSDMD-dependent pyroptotic ICD, with cancer-type-specific regulators (FTO-m6A, ERRα-HIF-1α, HPV E6/E7-Foxm1/KIF23) governing the net outcome. In M2 tumor-associated macrophages, USP19-STAT6 signaling suppresses NLRP3 to promote IL-10/TGF-β/VEGF secretion, while in M1 macrophages and dendritic cells, NLRP3 activation enhances antigen presentation and CD8+ T cell priming. In CD8+ T cells, NLRP3 exerts dual functions — canonical IL-18→IFN-γ signaling supporting CTL activity and non-canonical inflammasome-independent transcriptional regulation of unknown significance. In cancer-associated fibroblasts, chronic NLRP3-driven IL-1β promotes ECM remodeling and immune exclusion.

Emerging evidence suggests that NLRP3 exerts diverse and sometimes opposing functions in tumor cells, macrophages, dendritic cells, and CD8+ T cells. While NLRP3 activation may enhance antigen presentation, cytokine production, and cytotoxic immune responses in certain immune compartments, it may also promote immune evasion and tumor progression when activated in malignant cells. Furthermore, recent studies indicate that NLRP3 may possess inflammasome-independent activities, including transcriptional regulatory functions in T cells, although the significance of these non-canonical mechanisms in tumor-infiltrating lymphocytes remains largely unexplored (**Figure 2**).

To resolve these complexities, future studies should integrate single-cell and spatial multi-omics technologies, including scRNA-seq, spatial transcriptomics, and spatial proteomics. Such approaches will enable comprehensive mapping of NLRP3 signaling across heterogeneous cellular populations within the same tumor and clarify how cellular context influences therapeutic responses. These insights will be essential for determining whether NLRP3 modulators should be administered systemically or delivered through cell type-targeted strategies, such as nanoparticle-mediated delivery to tumor-associated macrophages.

### 6.2 Biomarker strategies for NLRP3-targeted therapy

For NLRP3 modulators to enter clinical development in gynecological cancers, companion biomarker strategies are needed to guide patient selection (48). We propose a tiered biomarker framework:

Tier 1. Expression-based markers: NLRP3, ASC, caspase-1, IL-1β, and IL-18 transcript and protein levels in tumor biopsies. IHC for ASC speck formation (a hallmark of inflammasome activation) provides greater functional resolution than NLRP3 expression alone. Serum IL-1β and IL-18 levels may serve as non-invasive pharmacodynamic markers of target engagement.
Tier 2. Pathway activity markers: Phospho-STAT3, phospho-NF-κB p65, and GSDMD N-terminal fragment levels as downstream readouts of NLRP3 pathway activation. Cleaved GSDMD IHC, in particular, distinguishes inflammasome-active from inflammasome-inactive tumors regardless of NLRP3 expression level.
Tier 3. Immune context biomarkers: Multiplex immunofluorescence and spatial profiling approaches to quantify CD8+ T-cell infiltration, FoxP3+ regulatory T cells, M1/M2 macrophage ratios, and PD-L1 expression in relation to NLRP3-positive cells.
Tier 4. Genetic biomarkers: NLRP3 polymorphisms (e.g., rs10754558) and somatic alterations in inflammasome-related genes that may influence individual NLRP3 signaling capacity and therapeutic responsiveness.

Future clinical trials evaluating NLRP3-targeted therapies in gynecological cancers should incorporate these biomarker assessments in both pre-treatment and on-treatment specimens, including tumor biopsies and blood-based samples. Such efforts will facilitate retrospective identification of responders and support refinement of predictive biomarker models. More broadly, artificial intelligence-driven knowledge graph approaches are emerging as powerful tools for integrating multi-dimensional clinical and molecular data to support personalized therapeutic decision-making (49).

Several important knowledge gaps also warrant further investigation. The temporal dynamics of NLRP3 activation during disease progression and treatment remain poorly understood (50). Longitudinal analyses of matched pre- and post-treatment samples may reveal how NLRP3 signaling evolves under therapeutic pressure and how these changes influence treatment outcomes. In addition, integrating NLRP3-related biomarkers with established predictors such as microsatellite instability (MSI), tumor mutational burden (TMB), and PD-L1 expression may improve patient stratification and enhance prediction of immunotherapy responses.

Looking ahead, two research directions appear particularly promising. Artificial intelligence and network pharmacology methodologies are increasingly capable of deconvoluting complex multi-target mechanisms—an approach that has been successfully applied to decipher traditional medicine formulations (51) and may prove valuable for understanding NLRP3’s pleiotropic functions. First, elucidating the crosstalk between ferroptosis and NLRP3-mediated pyroptosis may uncover combination strategies capable of enhancing immunogenic cell death and anti-tumor immunity. Second, the development of context-specific and tumor-targeted NLRP3 modulators, guided by biomarker-driven patient stratification, may enable precision immunotherapy approaches for gynecological malignancies. Translating these mechanistic insights into clinically actionable therapeutic strategies will be essential for improving patient outcomes.

## 7 Conclusion

NLRP3 functions as a double-edged sword in gynecological cancers, promoting immune suppression through PD-L1 upregulation and M2 macrophage polarization in ovarian cancer, exhibiting subtype-dependent effects in endometrial cancer, and being suppressed by HPV oncoproteins in cervical cancer. Meanwhile, its pyroptotic function can enhance chemotherapy sensitivity and activate anti-tumor immunity. These context-dependent roles underscore that therapeutic strategies targeting NLRP3 must be precisely tailored to tumor type, molecular subtype, and immune context to achieve optimal clinical benefit.

## Glossary

CC: Cervical cancer
CN-high: Copy number-high
CN-low: Copy number-low
DAMPs: Damage-associated molecular patterns
dMMR: Mismatch repair-deficient
EC: Endometrial cancer
EMT: Epithelial-mesenchymal transition
ERRα: Estrogen-related receptor alpha
GSDMD: Gasdermin D
HGSOC: High-grade serous ovarian cancer
HPV: Human papillomavirus
ICB: Immune checkpoint blockade
ICD: Immunogenic cell death
ICIs: Immune checkpoint inhibitors
IDO: Indoleamine 2,3-dioxygenase
IL: Interleukin
MCC950: NLRP3 inhibitor
MDSCs: Myeloid-derived suppressor cells
MSI-H: Microsatellite instability-high
MSS: Microsatellite-stable
NF-κB: Nuclear factor kappa-B
NLRP3: NOD-like receptor protein 3
OC: Ovarian cancer
POLE: DNA polymerase epsilon
ROS: Reactive oxygen species
STING: Stimulator of interferon genes
TAMs: Tumor-associated macrophages
TMB: Tumor mutational burden
TME: Tumor microenvironment
Tregs: Regulatory T cells
USP19: Ubiquitin-specific protease 19
VEGF: Vascular endothelial growth factor
